# Evaluation of cytotoxic, anti-oxidant, and apoptotic effects of *Dysphania botrys* extract on B16F10 and MCF-7 cell lines

**DOI:** 10.22038/ijbms.2025.87553.18912

**Published:** 2026

**Authors:** Fatemeh Forouzanfar, Elham Ramazani, Mohammad Esmaeili, Seyed Ahmad Emami, Zahra Tayarani-Najaran

**Affiliations:** 1 Neuroscience Research Center, Mashhad University of Medical Sciences, Mashhad, Iran; 2 Department of Neuroscience, Faculty of Medicine, Mashhad University of Medical Sciences, Mashhad, Iran; 3 Department of Biology, Yazd University, Yazd, Iran; 4 Medical Toxicology Research Center, Pharmaceutical Technology Institute, Mashhad University of Medical Sciences, Mashhad, Iran; 5 Department of Traditional Pharmacy, School of Pharmacy, Mashhad University of Medical Sciences, Mashhad, Iran; 6 Targeted Drug Delivery Research Center, Pharmaceutical Technology Institute, Mashhad University of Medical Sciences, Mashhad, Iran

**Keywords:** Amaranthaceae, Anti-oxidant, Apoptosis, Cytotoxicity, Dysphania botrys, Flavonoid content, Phenolic content

## Abstract

**Objective(s):**

*Dysphania botrys *(L.) Mosyakin & Clemants (Basionym: *Chenopodium botrys* L.), belonging to the Amaranthaceae family, has been used for the treatment of inflammation, bacterial and viral infections, and diabetes. In this study, we aimed to evaluate the potential cytotoxic, anti-oxidant, and apoptotic activities of methanol (MeOH) extract and petroleum ether (PE) and dichloromethane (DCM) fractions of *D. botrys* against B16F10 and MCF-7 cell lines.

**Materials and Methods:**

The anti-oxidant activities of fractions were measured using FRAP, DPPH, and β-carotene assays. The cytotoxicity of extracts and the intracellular ROS content were assessed using resazurin and DCFH-DA assays, respectively. A flow cytometry assay using PI staining was performed to measure the apoptotic activity of the fractions. Total phenolic and flavonoid content was determined using spectrophotometric methods.

**Results:**

The DCM fraction of *D. botrys* exhibited the highest anti-oxidant activity in FRAP, DPPH, and β-carotene assays, which also showed the highest amount of phenolic and flavonoid content compared to the MeOH extract and PE fraction. Cell viability and intracellular ROS content were significantly decreased following the treatment of B16F10 and MCF-7 cells with 100 and 200 µg/ml DCM and PE fractions. Treatment with 200 µg/ml DCM and PE fractions increased apoptosis in B16F10 cells.

**Conclusion:**

DCM fraction of *D. botrys* had significant anti-oxidant effects that may be associated with its phenolic and flavonoid compounds. It seems that terpenoid compounds are responsible for cytotoxic effects. Hence, complementary studies are needed to assess other bioactive compounds of *D. botrys *and their protective mechanisms.

## Introduction

Medicinal plants and their bioactive compounds provide numerous health benefits and are vital to the global economy. These bioactive metabolites are regarded as essential sources for the pharmaceutical, food, and cosmetic industries (1, 2). In recent decades, considerable attention has been given to the revival of medicinal plants as promising medicinal sources in modern medicine. This was due to the side effects of chemical drugs, the low cost of plant-derived drugs, and the resistance of some pathogens to chemical medicines (3, 4). Given the fundamental role of medicinal plants in complementary and alternative medicine, it seems necessary to identify, explore, and assess medicinal plant species at morphological, phytochemical, medicinal, and molecular levels (5). 

Cancer is one of the most important causes of death in the world. Two more common types of cancer are breast cancer and melanoma, with noteworthy risk associations (6). Based on numerous pieces of evidence, the most common cancer among women is breast cancer, which was globally responsible for 684,996 deaths in women (7). Also, melanoma is the most serious type of skin cancer that occurs in melanocytes and is known as one of the deadliest forms of skin cancer, with a death toll of nearly 75% (8). Several studies have strengthened the assumption that there may be an overlap between pathways affecting the development of breast cancer and melanoma. Hence, breast cancer survivors appear to have a higher risk of developing melanoma, and vice versa (9). Therefore, it seems essential to evaluate the association between these cancers and their possible treatments. 

*Dysphania* R.Br*.*, belonging to the family Amaranthaceae, includes a variety of weedy plants and comprises 46 species distributed in many regions of Europe, Asia, India, China, and North and South America (3, 10-11). *Dysphania* species are widely used in traditional medicine for their pharmacological properties and preclinical applications (12).* Dysphania botrys* (L.) Mosyakin and Clemants (Basionym: *Chenopodium botrys* L.) is native to Eastern, Central, and Southern Europe, Mongolia, and the Central Himalaya (13). In Iran, it is distributed in Azerbaijan, Hamedan, Mazandaran, Khorasan, Sistan and Baluchistan, and Tehran provinces (14). *D. botrys* contains mainly flavonoids, alkaloids, and terpenoids (13). One of the most important alkaloids of *D. botrys* is betaine, which is isolated from all parts of the stem and root of this plant (13). The essential oil is dominated by monoterpenes and sesquiterpenes, ranging from 0.08 to 2% in the plant aerial parts (15). Previous studies have also confirmed traditional uses of *D. botrys* in the treatment of a wide range of disorders (13). For example, in Iran, *D. botrys* has been used as an expectorant, anti-convulsant, and anti-asthma (13, 14). In Serbia, the dried aerial parts of *D. botrys* were found to have potent diuretic, antispasmodic, and anti-diarrheal activities (13). In traditional Indian medicine, *D. botrys *is mainly prescribed as a stimulant, diuretic, and antispasmodic agent. It has been used to treat asthma, cataracts, stomach, and liver diseases (13). Based on recent studies, anti-microbial, anti-inflammatory, anticancer, and anti-oxidant properties have also been reported (16-18). 

In the current study, we aimed to determine the cytotoxic and anti-apoptotic effects of the methanol (MeOH) extract and the petroleum ether (PE) and dichloromethane (DCM) fractions of *D. botrys *against B16F10 and MCF-7 cancer cells, and to evaluate their anti-oxidant activity using FRAP, DPPH, and β-carotene assays. 

## Materials and Methods

### Reagents and chemicals

DCM, MeOH, ethyl acetate (EtOAc), and PE were purchased from Dr. Mojallali (Tehran, Iran). The fluorescent probe propidium iodide (PI), Triton X-100, AlamarBlue (resazurin), and DCFH-DA (2′, 7′-Dichlorofluorescin diacetate) were purchased from Sigma (St Louis, MO, USA), and RPMI-1640 and fetal bovine serum (FBS) were bought from Gibco (Grand Island, USA).

### Plant materials

The aerial parts of *D. botrys* were collected in mid-summer 2020 from Shurgasht village, Neishabur, Razavi Khorasan, Iran, identified by Mrs. Mitra Souzani at Mashhad University of Medical Sciences (voucher No. 13601), and kept in the Herbarium of School of Pharmacy, Mashhad University of Medical Sciences, Mashhad, Iran. The plant aerial parts were dried in shade, powdered, and maintained in a dark place at room temperature.

### Plant extraction

To obtain crude MeOH extract, plant aerial parts (200 g) were extracted with MeOH (1.5 L) by the percolation method at room temperature, and the solvent was evaporated under reduced pressure at 40–45 °C by a rotary evaporator (Heidolph, Germany) and then freeze-dried (Operon, South Korea). The extract was suspended in 95% MeOH and fractionated successively by PE and DCM. Ultimately, the obtained fractions were dried using a rotary evaporator and freeze dryer. The extract and fractions were stored at -20 °C until use. To prepare stock solutions, the extract and fractions were diluted to 20 µg/ml in DMSO.

### 2, 2′-diphenyl-1-picrylhydrazyl radical (DPPH) assay

According to the protocol of a previous study, the DPPH free radical method was used to evaluate the anti-oxidant activity of the extract and its fractions. At first, 150 µl of MeOH extract and PE and DCM fractions of *D. botrys *(2.5-200 µg/ml) were exposed to 150 µl of 0.1 mM solution of DPPH in MeOH. The samples were incubated in the dark for 30 min at room temperature, and absorption was measured at 517 nm using a UV-2550 spectrophotometer (Shimadzu, Japan). MeOH was considered as blank (19).

### Ferric reducing anti-oxidant power (FRAP) assay

According to Sadeghi *et al*., the FRAP assay was used to assess the anti-oxidant capacity of the extracts based on Iron reduction. For the preparation of the stock solution, 5 ml of 10 mM 2, 4, 6-tris(2-pirydyl)-s-triazine (TPTZ) solution in 40 mM hydrochloric acid (HCl) was mixed with 50 ml of 300 mM acetate buffer (pH 3.6), and 5 ml of 20 mM ferric chloride hexahydrate (FeCl_3_ · 6H_2_O) solution. For the preparation of fresh FRAP reagent, 2.5 ml TPTZ solution, 25 ml acetate buffer, and 2.5 ml FeCl_3_ · 6H_2_O solution were mixed. Then 20 µl of MeOH extract and PE and DCM fractions of *D. botrys *(2.5–200 µg/ml) were added to 180 µl of FRAP reagent and poured into 96-well plates. The plates were incubated at 37 °C in the dark for 30 min, and the absorbance was measured at 595 nm using a UV-2550 spectrophotometer (Shimadzu, Japan). Distilled water was considered as blank, and FeSO_4_ · 7H_2_O (100-1000 μM) was used for calibration. The results were expressed as mM Fe^2+^/mg sample (20).

### Linoleic acid/β-carotene bleaching anti-oxidant assay

The anti-lipid peroxidation activity, known as total anti-oxidant capacity, of the extract and fractions was estimated following the methodology described by Nickavar and Esbati (21). In the first step, to prepare the stock solution, 0.5 mg of β-carotene was dissolved in 10 ml of chloroform, then mixed with 20 µl of linoleic acid and 200 mg of Tween 80. Then, after evaporation of chloroform under vacuum, 100 ml of aerated distilled water was added to form an emulsion. Afterward, 500 µl of the MeOH extract and the PE and DCM fractions (2.5-200 µg/ml) were added to 450 µl of the aforementioned mixture, which was incubated in a hot water bath at 50 °C for four hours. The absorbance was measured at 490 nm against a blank devoid of β-carotene using a UV-2550 spectrophotometer (Shimadzu, Japan) (22, 23).

### Total phenolic content assay

In our recent study, the Folin-Ciocalteu reagent was used to determine the total phenolic content of the extract and its fractions. At first, 100 µl of the MeOH extract and the PE and DCM fractions of *D. botrys *(2.5-200 µg/ml) were added to 200 µl of Folin-Ciocalteu reagent (diluted 1:10 with distilled water) and incubated in the dark for 5 min. Afterward, aqueous Na_2_CO_3_ (200 µl, 7% w/v) was added to the diluted solution, which was incubated again for two hours in the dark, and the absorbance was determined using a UV-2550 spectrophotometer (Shimadzu, Japan) at 765 nm. The calibration curve of gallic acid standard solutions was obtained in the same manner, and the resulting calibration equation was used to determine total phenolic content (24-26). 

### Total flavonoid content assay

The flavonoid content of the extract and fractions was measured according to the protocol of Farahmandfar *et al*. (2019), with some modifications. First, 500 µl of MeOH extract and PE and DCM fractions of *D. botrys *(2.5-200 µg/ml) were dissolved with 50 µl of 5% sodium nitrite (NaNO_2_) in MeOH for 6 min. Then, 50 μl of 10% aluminum chloride (AlCl_3_) and 100 μl of 4% sodium hydroxide (NaOH) were added, vortexed for one minute, and incubated at room temperature for 10 min thereafter. After 30 min, the flavonoid content was measured at 415 nm using a UV-2550 spectrophotometer (Shimadzu, Japan). Due to the equation obtained from the standard quercetin calibration curve, total flavonoid content was measured as mg of quercetin equivalent (27). 

### Cell culture

Two cancer cell lines, including MCF-7 (human breast cancer cells) and B16F10 (murine melanoma cells), were maintained in RPMI 1640 medium supplemented with 10% (v/v) fetal bovine serum (FBS) and 1% (v/v) penicillin-streptomycin. The cells were incubated in a humidified atmosphere (90%) with 5% CO_2_ at 37 °C. For each concentration- and time-course study, doxorubicin was used as a positive control; an untreated control sample was also included. The culture medium was replaced with fresh medium every two days. When cells reached 70–80% of confluence, they were exposed to different concentrations of each sample (28). 

### Cell viability assay

MCF-7 and B16F10 cells (10^4^ cells per well) were seeded in 96-well plates for 24 hr and then treated with MeOH extract and PE and DCM fractions (2.5-200 µg/ml) for 24 hr. They were then incubated for six hours after the addition of 20 µl (10 mg/ml) resazurin to each well, and the absorbance was measured by an ELISA microplate reader (Awareness, USA) at 570 and 600 nm (29).

### Intracellular ROS assay

Based on our previous study to evaluate the level of reactive oxygen species (ROS), MCF-7 and B16F10 cells (10^4^ cells per well) were seeded in 96-well plates and treated with MeOH extract and PE and DCM fractions (2.5-200 µg/ml) for 24 hr. The cells were treated with 24 mM H_2_O_2_ for 30 min, and exposed to 10 μl of 2′,7′-dichlorofluorescein diacetate (DCFH-DA) and incubated for 30 min. Finally, ROS levels were evaluated by measuring the fluorescent intensity of DCF at 504 nm (excitation) and 524 nm (emission) using a Synergy H4 Hybrid Multi-Mode Microplate Reader (BioTek, USA) (30). 

### Flow cytometry assay by propidium iodide staining

PI staining was used to measure the percentage of apoptotic cells. B16F10 cells (5×10^5^ cells per well) were seeded in 12-well plates and incubated for 24 hr. The cells were treated with 200 µg/ml of MeOH extract, PE, and DCM fractions, and a positive control for 24 hr. They were then washed, harvested, and incubated with 200 μl of hypotonic buffer (50 μg/ml PI in 0.1% sodium citrate and 0.1% Triton X-100) at 4 °C in the dark for 30 min before flow cytometric analysis (BD Biosciences, USA) (31). 

### Statistical analysis

All data are shown as mean ± SD of three independent assessments. One-way analysis of variance (ANOVA), Dennett’s *post hoc* test, and two-way ANOVA were performed using Graphpad Prism version 8.00. Comparisons were made relative to untreated controls, and *P<0.05, **P<0.01, and ***P<0.001 were considered statistically significant.

## Results

### DPPH scavenging capacity

**Figure 1:**
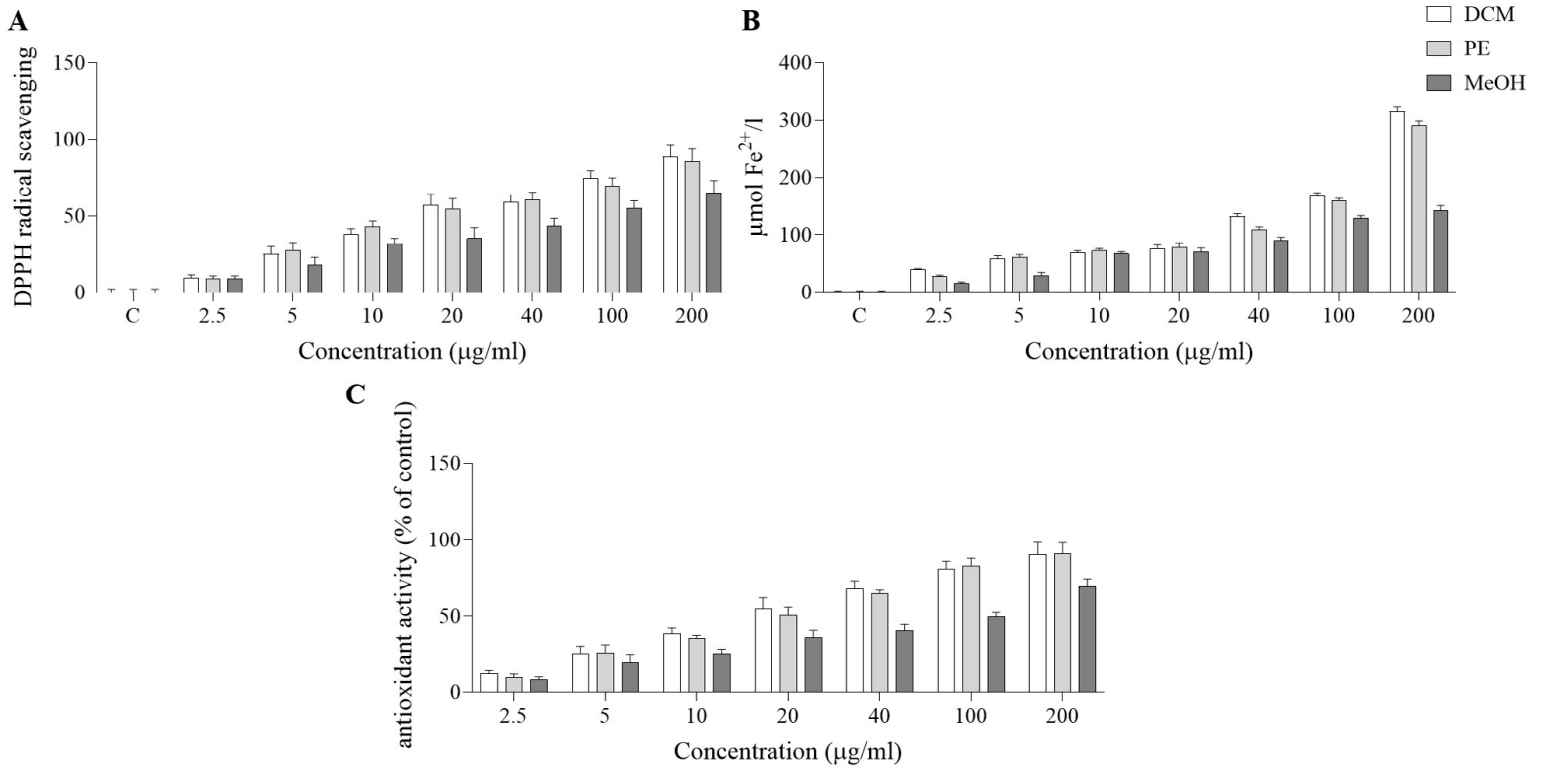
DPPH and FRAP free radical scavenging activities and linoleic acid/β-carotene antioxidant capacity of MeOH extract and DCM and PE fractions of *Dysphania botrys *(2.5-200 µg/ml)

Figure 1A shows the radical-scavenging effect of MeOH extract and PE and DCM fractions of *D. botrys*. Based on the results, the 200 µg/ml DCM fraction showed the highest anti-oxidant activity (88.68%), while the 200 µg/ml PE fraction and the MeOH extract showed scavenging activities of 85.85% and 65.03%, respectively.

### FRAP scavenging capacity

The results of the FRAP assay (Figure 1B) indicated that all analyzed samples exhibited noteworthy anti-oxidant effects. The anti-oxidant activity diminished in the order of DCM> PE> MeOH. DCM fraction (200 µg/ml) of *D. botrys* exhibited a value of 314.70 µmol Fe^2+^/l sample.

### Linoleic acid/β-carotene anti-oxidant capacity

To investigate anti-oxidant capacity, anti-lipid peroxidation activity was assessed relative to ascorbic acid. The result showed that all fractions displayed potent anti-oxidant activity. At 200 µg/ml, the DCM and PE fractions and the MeOH extract have higher anti-oxidant capacities (90.46%, 91.20%, and 69.485, respectively). At lower concentrations (2.5 µg/ml), the activity of DCM was higher (12.70%) than that of the PE fraction and MeOH extract (Figure 1C).

### Determination of total phenolic content

To determine overall anti-oxidant activity, the amount of phenolic content (mg gallic acid/g extract) was measured. As presented in Table 1, phenolic content ranged from 199.78 to 244.02 mg/g, and the DCM fraction exhibited the highest amount of phenolic content (244.02 mg/g) compared to the PE fraction and the MeOH extract.

### Determination of total flavonoid content

The results from the total flavonoid content test showed that the DCM fraction had the highest flavonoid content (114.38 mg/g), followed by the PE fraction and the MeOH extract. The range of flavonoid content (72.30–114.38 mg/g) is presented in Table 1.

### Effect of MeOH extract, DCM, and PE fractions on cell viability of MCF-7 and B16F10 cells

**Figure 2:**
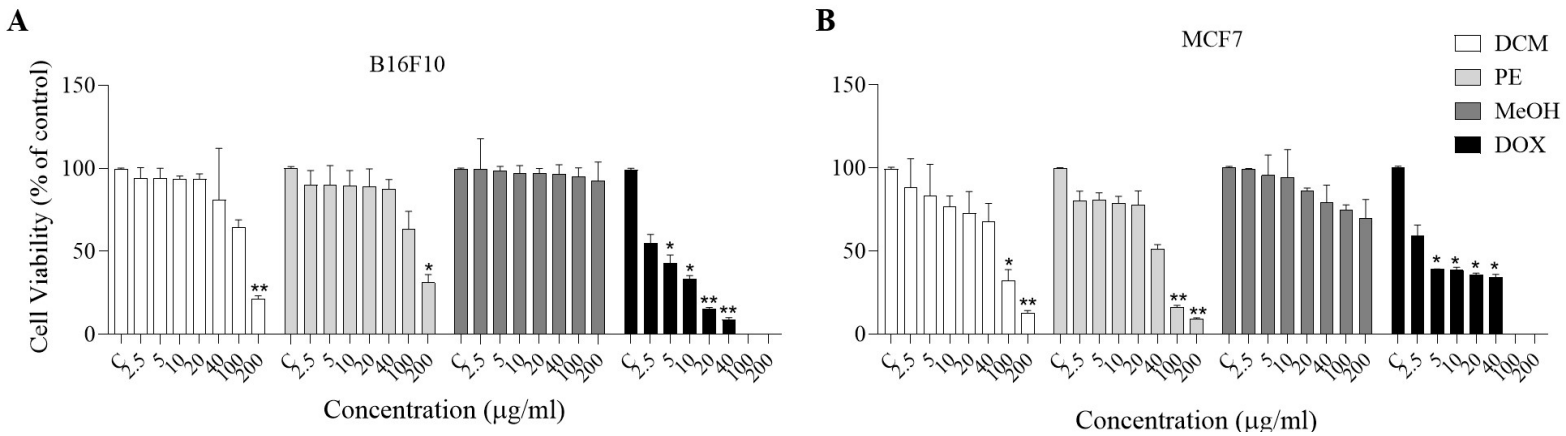
The effect of MeOH extract and DCM and PE fractions of *Dysphania botrys* (2.5-200 µg/ml) on the viability of A) B16F10 and B) MCF-7 cell lines

The cytotoxic effects of the MeOH extract and the DCM and PE fractions (2.5-200 µg/ml) on MCF-7 and B16F10 cells were clearly differentiated from the negative group using the resazurin assay. Based on the results, the cytotoxic effects of DCM and PE fractions were concentration-dependent. Incubating MCF-7 cells with 100 and 200 µg/ml DCM (*P*<0.05 and *P*<0.01) and PE (*P*<0.01) fractions for 24 hr leads to a significant reduction in cell viability. Also, treating B16F10 cells with 200 µg/ml of the DCM (*P*<0.01) and PE (*P*<0.05) fractions for 24 hr resulted in a noteworthy decrease in cell viability compared to the control group (Figure 2). 

### Effect of MeOH extract and DCM and PE fractions on MCF-7 and B16F10 cellular ROS levels

**Figure 3:**
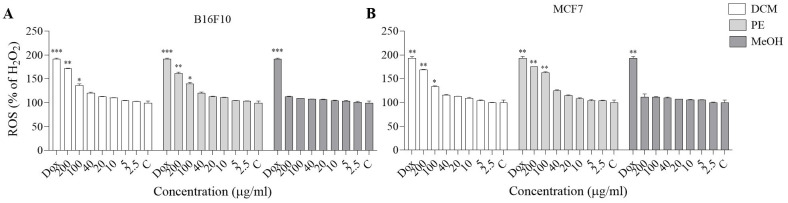
Effect of MeOH extract and DCM and PE fractions of *Dysphania botrys *(2.5-200 µg/ml) on cellular ROS level

The results showed that when MCF-7 and B16F10 cells were treated with H_2_O_2_, intracellular ROS levels were significantly elevated. Our results indicated that pretreatment with DCM and PE fractions at 100 and 200 µg/ml significantly suppressed ROS generation induced by H_2_O_2 _compared with the positive control (Figure 3).

### Effect of MeOH extract and DCM and PE fractions on B16F10 cellular apoptosis

**Figure 4:**
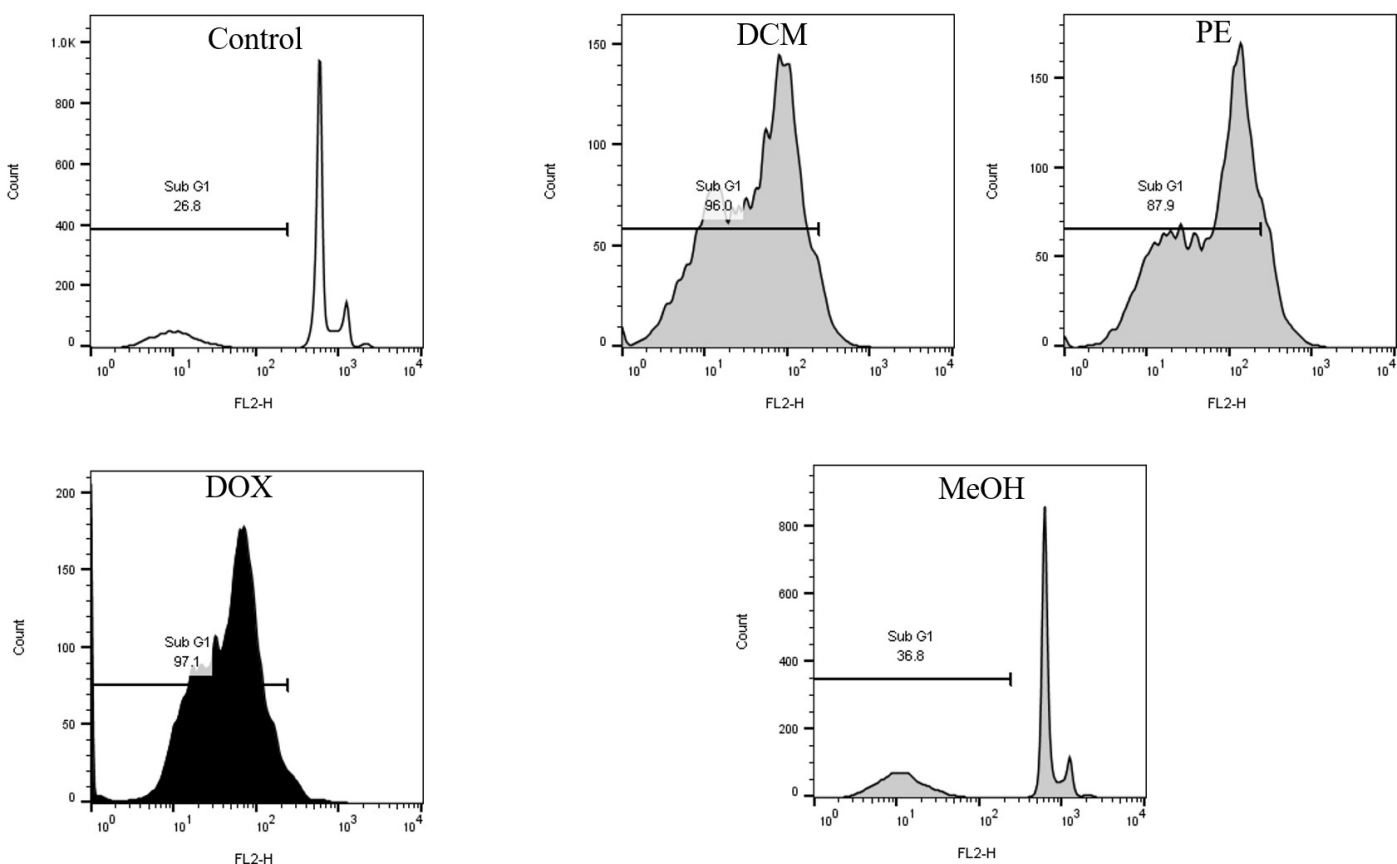
Effect of MeOH extract and DCM and PE fractions of *Dysphania botrys *B16F10 cellular apoptosis

Apoptotic effects of MeOH extract and DCM and PE fractions (200 µg/ml) on B16F10 were evaluated using the flow cytometry assay. The results confirmed that DCM and PE fractions can increase apoptosis, with apoptotic cell percentages of 96% and 87.9%, respectively. Untreated cells showed only 26.8% apoptotic cells (Figure 4). However, exposure of MCF-7 cells to 200 μg/mL of DCM and PE fractions did not induce apoptosis.

## Discussion

In this study, we investigated the cytotoxic and apoptotic activities of the MeOH extract and the DCM and PE fractions of *D. botrys* against B16F10 and MCF-7 cancer cell lines. We evaluated their anti-oxidant activities using various assays. There is substantial biochemical, biological, and clinical evidence that oxidative stress is involved in the development of various diseases, aging, and age-related diseases, as well as food spoilage (32). Anti-oxidants are chemical compounds that protect cells against free radical-induced damage. Therefore, assessing the anti-oxidant capacities of medicinal plants to achieve effective drugs is necessary (33). Our results indicate that the total phenolic and flavonoid contents in the DCM fraction of *D. botrys* were higher than in the PE fraction and the MeOH extract. Similarly, based on FRAP, DPPH, and β-carotene assays, the DCM fraction had higher anti-oxidant capacity than the PE fraction and the MeOH extract. These results have been confirmed by many other studies as well. The anti-oxidant and cytotoxic effects of lipophilic compounds of *Chenopodium berlandieri var. boscianum*, *Chenopodiastrum hybridum*, *Oxybasis rubra, *and *Oxybasis urbica* were explored. The results showed that all examined plants had the highest phenolic content in their herbs and seeds. In contrast, the herb extracts of *C. rubrum and C. urbicum* exhibited the best anti-oxidant activity among all extracts (34). Likewise, Ullah *et al*. reported that the chloroform, ethyl acetate, and *n*-hexane fractions of *D. botrys* exhibited radical-scavenging effects using the DPPH assay. Also, ethyl acetate fraction, chloroform fraction, and aqueous fractions of *D. botrys* showed the highest 2, 2-azinobis (3-ethylbenzothiazoline)-6-sulphonic acid (ABTS) free radicals scavenging activity (35). Similarly, in another study, Uddin *et al*. showed that the crude extracts and aqueous fractions of *D. botrys* presented noticeable DPPH scavenging effects (36). Moreover, the Kandsi *et al*. study indicates that the hydroethanol extract, ethyl acetate, and chloroform fractions of *D. ambrosioides* showed high anti-oxidant activity using DPPH, β-carotene bleaching, and FRAP assays (37). In addition, MeOH, ethyl acetate, and *n*-hexane extracts of *Chenopodium glaucum* revealed H_2_O_2_, ABTS, and DPPH scavenging activities (38). Furthermore, given the reported effects of flavonoids in previous studies, the anti-oxidant effects of *D. botrys* may be attributed to flavonoid compounds (39). The anti-oxidant activity of *D. botrys* is attributed to its compounds, such as phenols and flavonoids.

According to our results, concentration-dependent cytotoxicity was observed for the *D. botrys* extract and its fractions in B16F10 and MCF-7 cells, with greater cytotoxicity in the DCM and PE fractions. Also, all fractions of *D. botrys* increased ROS levels in a concentration-dependent manner. In contrast, at high concentrations of DCM and PE fractions, ROS generation was more noticeable, suggesting that it leads to oxidative stress and cell death. Excessive intracellular ROS production plays a critical role in a variety of abnormal pathological conditions and cell death. Therefore, these elements can be considered anticancer agents and may contribute to increased apoptosis of cancer cells (40, 41). Several research studies supported these findings. Rezaieseresht *et al*. reported that the essential oil from the aerial parts of *D. botrys *exerts anticancer effects by suppressing the growth of HeLa cells (18). No studies directly investigated the anticancer effect of *D. botrys *by increasing ROS levels. However, in our previous studies, we reported that increased ROS levels were involved in cancer cell death (42, 43). DCM and PE fractions of *D. botrys* may contribute to anticancer processes via decreasing cell viability and increasing ROS amount in B16F10 and MCF-7 cells.

Based on flow cytometry results using PI staining, we observed induction of apoptosis by DCM and PE fractions in B16F10 cells. It has been shown that treatment with *D. botrys *essential oil significantly increased the number of apoptotic HeLa cells in a dose-dependent manner, as assessed by flow cytometry and Annexin V-FITC/PI staining (18). Besides, the essential oil of *D. ambrosioides *has increased the apoptotic rate in SMMC-7721 cells (human liver cancer) in a concentration-dependent manner, as assessed by flow cytometry and Annexin V-FITC/PI staining (44). 

A) DPPH radical scavenging activity diminished in the order of DCM> PE> MeOH. B) Antioxidant activity diminished in the order of DCM> PE> MeOH. C) All fractions displayed potent antioxidant activity. All data are shown as mean ± SD of three independent assessments. Significance is shown as * *P*<0.05, ** *P*<0.01, and *** *P*<0.001 compared to the control group. DPPH: 2, 2′-diphenyl-1-picrylhydrazyl radical; FRAP: Ferric reducing antioxidant power; MeOH: Methanol; DCM: Dichloromethane; PE: Petroleum ether

**Table 1:** Total phenolic content (mg gallic acid/g extract) and flavonoid content (mg quercetin/g extract) of MeOH extract and DCM and PE fractions of *Dysphania botrys*

Fractions	Total phenolic content (mg gallic acid/g extract)	Total flavonoid content (mg quercetin/g extract)
DCM	244.02	114.38
PE	123.73	72.30
MeOH	199.78	90.26

MeOH: Methanol; DCM: Dichloromethane; PE: Petroleum ether

All data are shown as mean ± SD of three independent assessments. Significance is shown as * *P*<0.05, ** *P*<0.01, and *** *P*<0.001 compared to the control group.

MeOH: Methanol; DCM: Dichloromethane; PE: Petroleum ether

A) B16F10 and B) MCF-7 cell lines. All data are shown as mean ± SEM of three independent assessments. Significance is shown as * *P*<0.05, ** P<0.01, and *** *P*<0.001 compared to the positive group. MeOH: Methanol; DCM: Dichloromethane; PE: Petroleum ether; ROS: Reactive oxygen species

Treatment with 200 µg/ml DCM and PE fractions of *D. botrys* increased the percentage of apoptotic cells (96.0% and 87.9%) compared to untreated cells (26.8%). MeOH: Methanol; DCM: Dichloromethane; PE: Petroleum ether; ROS: Reactive oxygen species

## Conclusion

*D. botrys *and its phytochemicals have beneficial therapeutic effects and hold promise as treatments for a wide range of pathological conditions, including cancer. The results of the present study showed that the DCM fraction of *D. botrys *has more significant cytotoxicity and anti-oxidant properties than the PE fraction and MeOH extract. Therefore, it can be used as a potential treatment agent to slow cancer cell growth and promote cancer cell death. More extensive *in vivo* and clinical studies are needed to develop cancer drugs using this plant for the prevention or treatment of breast or skin cancer.

## Declaration

We have not used any AI tools or technologies to prepare this manuscript.
